# A cross-sectional analysis of air pollution in primary schools and children fatigue

**DOI:** 10.3389/fpubh.2025.1595089

**Published:** 2025-09-01

**Authors:** Vaida Taminskiene, Nina Prokopciuk, Vilmante Karvelyte, Egle Vaitkaitiene, Mindaugas Butikis, Algirdas Valiulis, Vilte Sapronaite, Gintare Talmontaite, Ginreta Megelinskiene, Karolina Sceliokiene, Rimantas Stukas, Arunas Valiulis

**Affiliations:** ^1^Department of Public Health, Institute of Health Sciences, Faculty of Medicine, Vilnius University, Vilnius, Lithuania; ^2^Clinic of Children’s Diseases, Institute of Clinical Medicine, Faculty of Medicine, Vilnius University, Vilnius, Lithuania; ^3^Medical Academy, Lithuanian University of Health Sciences, Kaunas, Lithuania; ^4^Department of Rehabilitation, Physical and Sports Medicine, Institute of Health Sciences, Faculty of Medicine, Vilnius University, Vilnius, Lithuania; ^5^Faculty of Medicine, Vilnius University, Vilnius, Lithuania

**Keywords:** children fatigue, air pollution, environmental health, particulate matter, primary school, dust aggregates, micro elemental composition

## Abstract

**Introduction:**

Childhood fatigue is influenced by various factors, including health status, socioeconomic conditions, lifestyle choices, and environmental factors like air pollution. In this study we aimed to explore the relationship between children’s fatigue and air pollution in the classrooms.

**Methods:**

547 children from eight primary schools were enrolled into the study. Air pollution was measured in the classrooms including concentration of particulate matter (PM1, PM2.5, PM10) and micro elemental analysis of dust. Fatigue was assessed by the Pediatric Quality of Life Inventory Multidimensional Fatigue Scale self-reports in scores ranging from 0 to 100. Higher scores indicated less fatigue. Multivariate linear regression was performed to explore factors independently associated with children’s fatigue.

**Results:**

Mean age (± standard deviation [SD]) of respondents was 9.03 (±0.42) years; 44.9% were males. The mean (±SD) total fatigue score was 80.13 (±7.99). We found that higher levels of fatigue in children were linked to worse overall health, lower academic performance, and fewer extracurricular activities. Additionally, levels of particulate matter, barium, and vanadium in the natural dust aggregates were independently related to increased fatigue.

**Conclusion:**

A cross-sectional type of our study only allows for the confirmation of statistical associations between fatigue levels and their possible determinants as specific air pollutants; further research is needed to explain and understand causal pathways better.

## 1 Introduction

Air pollution is a relevant public and environmental health issue which has a significant impact on population health (1, 2). The World Health Organization (WHO) indicates indoor air pollution as the largest environmental health risk factor which is responsible for more than 3 million premature deaths worldwide (3). Although majority of these deaths occur in developing countries, indoor air quality remains an important health factor at a global level (4). Particulate matter is one of the most important pollutants, causing oxidation and inflammation in tissues which lead to various chronic diseases and health conditions. Health effects of particulate matter depend not only on particle size, but also on composition. More than 15 percent of particulate matter consists of small amounts of various chemical elements (alkali, alkaline earth, transition and basic metals, semimetals, non-metals, halogens and lanthanides) (5). The composition of these elements can vary depending on the environment and accordingly affect health.

Air pollution affects the health of all people in all age groups, including an increase in perinatal disorders, infant mortality, chronic diseases such as respiratory, cardiovascular and mental disorders, type 2 diabetes and other health conditions (6, 7). Infants and children are one of the most sensitive population groups to air pollution, since they get higher air intake per body weight and their organs are still developing (8). Higher children exposure to air pollution might be related to reduced lung function, increase of respiratory disorders including asthma, neurodevelopmental disorders, cancer, and higher risk of chronic diseases in adulthood (8, 9). Exposure to toxic heavy metals in polluted air impairs children’s cognition, learning, may worsen fatigue and emotional well-being, can lead to increased vulnerability to mental health disorders like depression (10–12). The mechanism includes heavy metal accumulation in the prefrontal cortex, leading to neuroinflammation and disrupted neurotransmitter balance, and impaired brain network functioning (12, 13). Studies have identified significant associations between blood levels of lead, zinc, arsenic, selenium, mercury, and manganese and mental health issues in children, including conduct problems, learning disabilities, anxiety, and impulsivity–hyperactivity (14), as well as between elevated concentrations of strontium and barium and an increased risk of depression in adults (15). Although the exact mechanisms of barium toxicity remain unclear, its interference with potassium channel function and membrane ion regulation suggests a potential pathway for neurophysiological disruption, raising concerns about its impact on the developing nervous system and mental health in children (16). Prenatal vanadium exposure is associated with impaired neurodevelopmental outcomes in children, including reduced mental development scores, increased risk of developmental delay, and higher risk of attention deficit hyperactivity disorder (17, 18). Despite growing concern, research on the neurochemical pathways linking heavy metal exposure such as barium or vanadium to mental health in children remains limited.

Indoor air quality at schools plays an important role (10, 19, 20). Children spend a significant part of their time at schools, mostly in the classrooms where air pollution might be higher compared to their homes (10, 20). While there are many pieces of evidence proving air pollution association with chronic diseases (6–9,), and our previous research revealed that higher concentrations of vanadium may be associated with more frequent acute respiratory infections, there is still lack of data about minor health effects of air pollution in relatively safe environments where most children spend significant part of their time. Health outcomes as effects on intellectual functioning and performance, mental processes and fatigue might be more difficult to measure and there are less studies focusing on it. Fatigue is a common sign among school age children which often remains underestimated (21). Fatigue in children can be determined by multiple factors: general health condition, workload at school, lifestyle and psychological factors, socioeconomic and physical environment as well as the quality of the air they breathe (22–26). Previous studies have linked common air pollutants such as particulate matter and O_3_ to increased fatigue (24). The study from the United States indicated higher levels of lethargy in children on days when they were exposed to higher levels of carbon monoxide and nitrogen dioxide (11). However, there is insufficient data on how the specific composition of dust, particularly the concentrations of individual elements and metals, in typical, everyday environments may affect children’s overall well-being and levels of fatigue.

Gaining a deeper understanding of main air pollutants physiological effects is crucial (2, 5), including the impact of every day exposure on well-being and cognitive function. Therefore, in this study aimed to describe associations between children fatigue and air pollution in the classrooms including elemental composition of the dust.

## 2 Materials and methods

**Figure 1:**
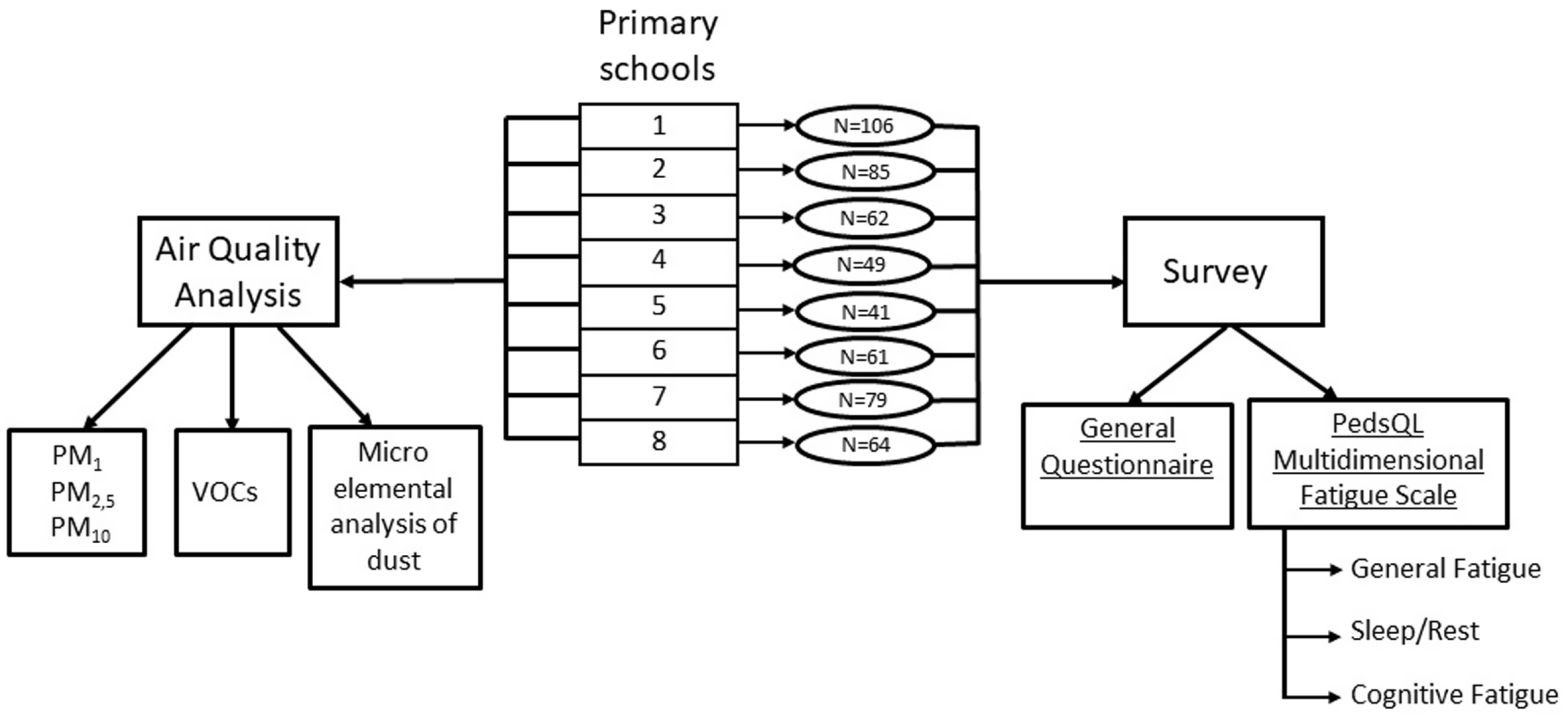
Flowchart of study design.

A cross-sectional study was performed in 8 primary schools in Vilnius, Lithuania. Vilnius has an average annual temperature of 7.0°C with 640 mm of precipitation, and is predominantly influenced by southwest and west winds carrying air masses from central and eastern Europe (27). Study composed of two parts: measurement of air pollution in the classroom and fatigue assessment of children (Figure 1). Invitations to participate in the study analyzing air pollution were sent to 107 schools in Vilnius. From the 25 schools that consented to participate in the study, every other school was selected in random order. Due to absence of primary classes, one school was excluded from the sample. From 11 school selected, 8 agreed to participate in the survey. Each school enrolled in the study was assigned a number from 1 to 8 to ensure data protection and confidentiality. Schools numbered 1, 4, 6, and 7 were located in the central area of the city, schools numbered 2, 3, 5, and 8 were located in the peripheral part. All schools were located no more than 250 meters from roads, highways, or railways. Data of the study were collected during the 2021/2022 winter season. The study was approved by the Vilnius Regional Biomedical Research Ethics Committee (protocol code 2021/11-1,381-862). Consents from schools’ administrations as well as assents from children to participate in the study were obtained.

### 2.1 Measurement of air pollution in schools

Air pollution was measured in the typical/standard classrooms of schools enrolled into the study. According to the applicable hygiene standards, classroom ventilation can be ensured by both natural and mechanical means. In all classrooms there must be a possibility for natural ventilation through openable windows. Classrooms that are not equipped with a mechanical ventilation system must be ventilated after each lesson (28). During winter measurements in classrooms were made once in each studied school. In the schools, indoor measurements were conducted under the conditions typically found in classes during lessons from 8 am to 2 pm in five - eight classrooms on different floors for 10 min each. Generally, classroom windows were opened during breaks. Measurements of fraction particulate matter [PM_1_ (0.3–1 μm), PM_2.5_ (0.3–2.5 μm), PM_10_ (0.3–10 μm)], volatile organic compounds (VOCs) and micro elemental analysis of dust were performed. To determine aerosol particle number concentration (PNC) and particle mass concentration (PMC) in primary schools, an optical particle sizer (OPS, TSI model 3,330, PNC in size range of 0.3–10.0 μm) were used. The PMC was calculated by OPS software with the predefined particle density of 1 g/cm^3^. Before measurements, the instruments were checked for contamination by using high-efficiency particulate arrestance filters. In parallel with the OPS, a Dräger X-am 8,000 (Drägerwerk AG & Co. KGaA, Germany) gas detector was used to determine the concentration of VOCs.

Collecting aerosol samples for trace element analysis in classrooms is challenging, as obtaining a sufficient sample mass often requires a prolonged period. Despite the fact that measurements on filters have advantages (29)—we can measure the concentrations of heavy metals in real time, it has an important disadvantage—the sample weight is small, so as a rule, the measurement errors of trace elements are large. Consequently, we opted to use dust samples instead, which typically accumulate in areas that are not reached during routine classroom cleaning. Aerosol particles can coagulate and deposit in the form of dust; thus, it is most convenient to study the micro elemental composition of the aerosols by collecting dust samples where they naturally accumulate. Such places in the classrooms not usually subjected to wet cleaning are the surfaces of high cupboards and places behind the radiator heaters where aerosol particles are deposited due to thermophoretic forces (30). Dust samples representing aerosol pollution were collected from the places behind radiators in each classroom of the primary school. It was avoided to collect dust samples from the floor due to the impact of soil contamination brought into the classroom by shoes.

Vacuum cleaner with an analytical filter type FPP (Filtering Polymeric Fibrous Materials) was used for dust collection. Plastic boxes (60 mL) were filled with collected dust samples. A micro elemental analysis of aerosol pollution was carried out using a SPECTRO XEPOS (Spectro Analytical Instruments GmbH, Germany) energy dispersive X-ray fluorescence (ED-XRF) spectrometer at the Lithuanian Geological Survey. The concentrations of arsenic (As), barium (Ba), bromine (Br), chromium (Cr), copper (Cu), manganese (Mn), nickel (Ni), lead (Pb), rubidium (Rb), antimony (Sb), tin (Sn), strontium (Sr), vanadium (V), tungsten (W), zinc (Zn), and zirconium (Zr) were measured in dust samples. Our study did not include radioactive elements, focusing instead on heavy metals detectable by our equipment. We analyzed potential correlations between heavy metals and self-reported fatigue. Some heavy metals (Hg, Co, Cd) were below the detection limit and therefore not included in the study. Notably, research on the health effects of trace elements in children remains limited, and while Lithuania has indoor air limits for heavy metals, they are not specifically tailored to children. The measurement time of one sample was 600 s and the accuracy of elemental composition was less than 10%. A more detailed methodology can be found in our previous publication (9).

### 2.2 Fatigue assessment

Age-appropriate versions of PedsQL Multidimensional Fatigue Scale proxy reports were used to assess the fatigue experienced by the children (31). Fatigue scale consisted of the 18 questions divided into three equal subscales (General Fatigue, Sleep/Rest and Cognitive Fatigue). The General Fatigue subscale includes 6 questions about feelings of tiredness and weakness, not being able to do things you enjoy or spend time with friends, and difficulties finishing or starting chores. The Sleep/Rest subscale includes 6 questions about sleep duration, difficulty staying asleep through the night, feeling tired in the morning, frequency of naps, rest, and time spent in bed. The Cognitive Fatigue questions aim to determine the child’s difficulties to concentrate, remember what is said to him/her, what he/she hears or thinks, the speed of thinking, and the ability to remember several things at once (32). Children assessed their experienced fatigue in different areas in points from 0 (never) to 4 (almost always). Scores for each question were recalculated to a 100-point scale according to the authors’ instructions. Higher scores correspond to lower levels of fatigue. Mean scores for each subscale as well as the Total Fatigue score were calculated. Children’s fatigue was assessed no later than 3 days after the air pollution measurements were taken.

Children also were asked to complete a general questionnaire which was created by authors and provided descriptive characteristics about study participants: gender, age, height and weight, health and living conditions and other details.

### 2.3 Statistical analysis

Descriptive statistics were performed. In univariate analysis mean fatigue score was compared between groups using independent t-test. A one-way ANOVA was conducted to compare fatigue scores in schools, additionally *post-hoc* Bonferroni test (33) was performed to identify which specific schools have significant differences between their mean fatigue scores. Spearman correlation was used to assess the associations between fatigue scores and air pollution or other possible risk factors. Linear regression models were created to identify which factors were independently associated with children’s fatigue. Two models were created for overall fatigue score as well as each separate scale. The first model included all variables significantly related to fatigue score and the second one included only air pollution indicators.

Additionally, composition of air quality in schools with the highest and the lowest mean fatigue scores was analyzed. IBM SPSS statistics for Windows, version 28 was used for statistical analysis. A *p*-value of <0.05 indicated statistically significant difference.

## 3 Results

Eight primary schools located in different areas of Vilnius city were enrolled into the study. Air pollution including elemental composition of dust was measured in the classrooms. The subjective fatigue level of 547 children who study in these classrooms was assessed.

Primary school students aged 8 to 10 years were included in the survey. The mean age (±SD) of respondents was 9.03 (±0.42) years. More girls (55.1%) than boys participated in this study. Most children assessed their general health as good or very good. Over 90% of children participated in extracurricular activities, while just over one-third assessed their academic performance as good or excellent. Further characteristics of study participants are presented in Table 1.

**Table 1:** Characteristics of the study participants.

Parameters		Total (N = 547)
% Males (*n*)		44.9 (244)
Mean age (±SD)		9.03 (±0.42)
Mean body mass index (BMI) (±SD)		16.89 (±3.02)
Mean BMI–z score (±SD)		−0.0013 (±1.00)
Child’s general health	% very good (n)	37 (201)
	% good (n)	43.6 (237)
	% moderate (n)	16.2 (88)
	% satisfactory, (very) poor (n)	3.1 (17)
Number of siblings	% none (n)	13.8 (75)
	% one (n)	58.9 (321)
	% two (n)	21 (115)
	% three or more (n)	6.2 (34)
Learning results at school	% very good and good (n)	38.4 (209)
	% moderate (n)	60.1 (327)
	% poor and very poor (n)	1.5 (8)
Number of afterschool activities	% none (n)	9.4 (51)
	% one (n)	45.3 (247)
	% two or more (n)	45.4 (247)
Housing type	% house (n)	30.4 (164)
	% cottage (n)	8 (43)
	% apartment (n)	61.6 (332)
% live in bedroom alone, without siblings (n)		48 (262)
Mean number of hours spent outdoors in autumn (±SD)		2.13 (±1.03)
Mean number of hours spent outdoors in winter (±SD)		2.19 (±1.03)
Mean number of hours spent outdoors in spring (±SD)		2.70 (±1.10)
Mean number of hours spent outdoors in summer (±SD)		3.65 (±0.91)

### 3.1 Fatigue level of children

Children were asked to complete an age-appropriate version of the PedsQL Multidimensional Fatigue Scale and self-assess their fatigue. In our study we found that children experience different signs of fatigue: they often feel tired, sleep and rest a lot, have difficulties thinking quickly, spend a lot of time in bed. The mean total fatigue score was 80.13 (±7.99). The highest score was for General Fatigue scale [81.15 (±9.99)] and lower scores were for Sleep/Rest [79.71 (±9.29)] and Cognitive Fatigue [79.51 (±10.39)] scales. There was no statistically significant difference between fatigue level in boys and girls. What is more, fatigue did not correlate with the age and BMI of respondents.

The mean overall fatigue scores differed in schools and varied from 77.86 (±7.05) to 81.89 (±7.35). *Post-hoc* comparison using Bonferroni test indicated that fatigue scores were significantly lower in the 1st school compared to the 6th (Table 2).

**Table 2:** The mean (±SD) fatigue scores of children in 8 schools.

School number (N)	1 (106)	2 (85)	3 (62)	4 (49)	5 (41)	6 (61)	7 (79)	8 (64)
Total Fatigue score*	77.86 (±7.05)*	81.18 (±8.29)	79.89 (±9.04)	81.48 (±8.03)	79.60 (±7.25)	81.89 (±7.35)*	80.39 (±8.90)	79.99 (±7.24)
General Fatigue score*	78.41 (±8.92)*	82.61 (±10.22)	80.74 (±11.31)	82.53 (±10.62)	79.94 (±8.19)	84.09 (±10.41)*	81.75 (±10.56)	80.35 (±8.57)
Sleep/Rest score*	77.52 (±9.23)*	80.79 (±9.64)	80.18 (±9.07)	82.95 (±8.37)*	79.39 (±9.65)	81.28 (±8.11)	77.69 (±9.94)*	80.14 (±8.87)
Cognitive Fatigue score	77.65 (±9.26)	80.19 (±10.62)	78.76 (±10.87)	78.74 (±10.90)	79.57 (±10.19)	80.25 (±10.64)	81.75 (±11.23)	79.45 (±9.70)

*p < 0.05.

### 3.2 Quality of the air in the classrooms

To assess the quality of the air in the classroom following indicators were measured: fractions particulate matter, VOCs, elemental composition of dusts (As, Ba, Br, Cr, Cu, Mn, Ni, Pb, Rb, Sb, Sn, Sr., V, W, Zn, Zr). Table 3 presents main air quality indicators in 8 primary schools.

**Table 3:** Air quality indicators measured in schools.

School number	1*	2	3	4	5	6*	7	8
PM_1_ μg/m^3^, mean	1.23 ↑↑	1.09	1.03	0.61	1.20	0.62 ↓↓	0.60	1.96
PM_1_ particles/m^3^, mean	18 ↑↑	17	17	9	19	9 ↓↓	11	50
PM_2.5_ μg/m^3^, mean	5.21 ↑↑	4.40	3.93	3.80	5.21	3.51 ↓↓	2.52	4.30
PM_2.5_ particles/m^3^, mean	19 ↑↑	18	18	10	20	10 ↓↓	12	51
PM_10_ μg/m^3^, mean	43.85 ↑↑	39.13	24.84	36.70	39.12	36.19 ↓	26.10	29.02
PM_10_ particles/m^3^, mean	20 ↑↑	18	19	10	21	10 ↓↓	12	51
VOCs, ppm	0.96 ↑↑	1.02	0.85	0.66	0.70	0.70 ↓↓	0.68	0.69
As, ppm	6.29 ↓	6.23	5.79	5.29	28.17	7.94 ↑↑	20.93	4.33
Ba, ppm	1,532 ↑↑	890	480	740	3,301	654 ↓↓	1,653	272
Br, ppm	14.93 ↓	17.36	51.55	10.15	20.14	9.64 ↓↓	19.07	49.04
Cr, ppm	96.21 ↓↓	89	147	97.1	64	127.63 ↑	180	168
Cu, ppm	134.12 ↑	108.63	138.79	69.12	436.0	42.63 ↓↓	77.83	134.60
Mn, ppm	72 ↓↓	56	319	91	152	87 ↓	159	240
Ni, ppm	8.4 ↓	11.47	61.06	6.41	14.11	5.74 ↓↓	20.46	26.88
Pb, ppm	17 ↓↓	24	114	19	132	34 ↓	504	185
Rb, ppm	11.81 ↓	11.69	28.94	12.41	20.64	7.52 ↓↓	18.52	23.53
Sb, ppm	7.52 ↓	11.48	10.07	5.63	9.48	4.83 ↓↓	13.0	10.74
Sn, ppm	6.11 ↓	7.45	6.17	4.84	6.24	5.71 ↓↓	9.42	9.42
Sr, ppm	82.69 ↓	63.0	135.0	77.15	170.0	52.70 ↓↓	136.0	110.08
V, ppm	41.63 ↑↑	12.69	20.06	38.42	19.07	29.53 ↑	17.18	15.49
W, ppm	11.85 ↓	11.58	21.39	8.56	19.48	7.91 ↓↓	14.69	34.61
Zn, ppm	319 ↓↓	510	1,251	424	17,865	384 ↓	1755	1887
Zr, ppm	27.42 ↓↓	32.0	122.0	30.42	104.00	31.80 ↓	93.55	120.0

↓↓ 1st quartile; ↓ 2nd quartile; ↑ 3rd quartile; ↑↑ 4th quartile. *Arrows next to the pollutant values at schools 1 and 6 show the relative position (quartile) of each concentration within the overall distribution across all schools.

Additionally, composition of air pollutants was compared in schools where fatigue scores were the highest (school no 6) and the lowest (school no 1). Table 3 includes arrows next to the pollutant concentration values for schools 1 and 6, indicating the quartile in which each value falls relative to the pollution levels determined for all schools participating in the study. These arrows help visualize how each measurement compares to the distribution of pollutant levels across all schools included in the analysis, providing a clearer understanding of whether a particular concentration falls within the lower, middle, or upper range of observed values. Concentrations of particulate matter in school no 1 were higher than the total median of this study measurements as well as concentrations of VOCs, Ba, Cu and V. In school no 6 only As, Cr and V were above median.

### 3.3 Association of increased fatigue level with air pollution and other possible determinants

Possible determinants of increased fatigue level in children were analyzed. In univariate analysis fatigue correlated with the general child’s health condition: *r* = 0.338 for Total Fatigue score; *r* = 0.329 for General Fatigue scale; *r* = 0.190 for Sleep/Rest scale and *r* = 0.303 for Cognitive Fatigue scale (*p* < 0.001). The number of siblings did not correlate with the children’s lethargy. Housing type as well as having a separate bedroom were not related to fatigue scores. Children with lower fatigue scores reported worse results at school:

- Total Fatigue scale: *r* = 0.174; *p* < 0.001.
- General Fatigue scale: *r* = 0.166; *p* < 0.001.
- Sleep/Rest scale: *r* = 0.134; *p* = 0.002.
- Cognitive Fatigue scale: *r* = 0.127; *p* = 0.003.

Only Cognitive Fatigue score correlated with the number of afterschool activities (*r* = 0.119; *p* = 0.005): children who attended more extracurricular activities felt less fatigue. The total time spent outdoors significantly correlated with Sleep/Rest scale (*r* = 0.086; *p* = 0.045) and Cognitive Fatigue scale (*r* = 0.114; *p* = 0.008).

As shown in Table 4, overall fatigue scores were negatively correlated with concentrations of PM_1_, PM_2.5_, PM_10_, Cu and V: higher pollutant concentrations were associated with higher fatigue levels. General Fatigue Scale scores were lower in schools where Cu and W amounts were higher; Sleep/Rest Scale scores correlated with As, Ba and Sr. Cognitive fatigue was higher in the classrooms with higher amounts of Pb and V.

**Table 4:** Correlations between air pollutants and PedsQL Fatigue scores.

Air pollutant	Total fatigue score	General fatigue scale	Sleep/rest scale	Cognitive fatigue scale
PM_1_ μg/m^3^, mean	−0,110**	−0,133**	−0,049	−0,078
PM_1_ particles/m^3^, mean	−0,123**	−0,146**	−0,084	−0,058
PM_2.5_ μg/m^3^, mean	−0,126**	−0,132**	−0,061	−0,087*
PM_2.5_ particles/m^3^, mean	−0,123**	−0,146**	−0,084	−0,058
PM_10_ μg/m^3^, mean	−0,078	−0,074	−0,036	−0,060
PM_10_ particles/m^3^, mean	−0,127**	−0,150**	−0,086*	−0,063
VOCs, ppm	−0,060	−0,047	−0,040	−0,038
As, ppm	−0,017	0,011	−0,095*	0,051
Ba, ppm	−0,059	−0,039	−0,110**	0,023
Br, ppm	−0,030	−0,045	−0,038	0,012
Cr, ppm	0,038	0,032	−0,016	0,047
Cu, ppm	−0,111*	−0,127**	−0,066	−0,062
Mn, ppm	0,009	−0,006	0,001	0,011
Ni, ppm	−0,023	−0,038	−0,042	0,021
Pb, ppm	0,064	0,055	−0,005	−0,089*
Rb, ppm	−0,044	−0,065	−0,028	−0,019
Sb, ppm	0,016	0,013	−0,050	0,075
Sn, ppm	0,005	−0,007	−0,057	0,071
Sr, ppm	−0,069	−0,076	−0,098*	0,009
V, ppm	−0,088*	−0,076	−0,048	−0,086*
W, ppm	−0,075	−0,095*	−0,076	−0,013
Zn, ppm	0,042	0,023	0,014	0,061
Zr, ppm	0,043	0,029	0,024	0,045

*p < 0.05; **p < 0.001.

In order to find out the most important factors independently associated with increased level of fatigue in children linear regression models were created. Variables which significantly correlated in univariate analysis model were included into regression.

As it can be seen from the Table 5, the most important fatigue determinant was subjective health assessment. An unstandardized B coefficient indicates that for each one-category improvement in health assessment [e.g., moving from “satisfactory/(very) poor” to “moderate”], the fatigue score increases by an average of 2.85 points, highlighting a positive association between better health assessments and higher fatigue scores (lower fatigue level). Overall fatigue scores were also higher in children with better grades at school. None of air quality variables were independently related to fatigue level in the first linear regression model, indicating their lower impact on fatigue level. The second model was created only with air quality indicators and showed that higher concentration of PM_1_ and vanadium were independently associated with lower fatigue score. Some additional correlations were found analyzing separate PedsQL Multidimensional Fatigue scales: higher barium concentrations were related to lower Sleep/Rest scale scores. Cognitive Fatigue scores were higher in children who attended more after-school activities and lower in children who were exposed to higher concentrations of PM_2.5_.

**Table 5:** Linear regression models describing factors independently associated with PedsQL fatigue scores.

Variables	Unstandardized coefficient	Standardized coefficients			
	B	Std. error	Beta	T	*p*-value
Total fatigue score, model 1					
Health	2.854	0.422	0.286	6.760	<0.001
Results at school	1.357	0.658	0.087	2.061	0.040
Constant	67.892	1.791		37.911	<0.001
*N* = 540; R^2^ = 0.103; Adjusted R^2^ = 0.099; *F* = 30.787; *p* < 0.001					
Total fatigue score, model 2					
V, ppm	−0.066	0.031	−0.091	−2.106	0.036
PM_1_ μg/m^3^, mean	−1.751	0.832	−0.091	−2.106	0.036
Constant	83.613	1.316		63.528	<0.001
*N* = 546; R^2^ = 0.014; Adjusted R^2^ = 0.010; *F* = 3.770; *p* = 0.024					
General Fatigue scale score, model 1					
Health	3.967	0.512	0.316	−7.750	<0.001
Constant	68.629	1.661		41.321	<0.001
*N* = 542; R^2^ = 0.100 Adjusted R^2^ = 0.098; *F* = 60.064; *p* < 0.001					
General Fatigue scale score, model 2					
PM_1_ μg/m^3^, mean	−2.434	1.026	−0.101	−2.373	0.018
Constant	83.719	1.162		72.049	<0.001
*N* = 546; R^2^ = 0.010; Adjusted R^2^ = 0.008; *F* = 5.633; *p* = 0.018					
Sleep/Rest scale score, model 1					
Health	1.399	0.512	0.120	2.729	0.007
Results at school	1.690	0.797	0.093	2.119	0.035
Ba, ppm	−0.001	0.001	−0.083	−1.960	0.050
Constant	72.428	2.271		31.895	<0.001
*N* = 540; R^2^ = 0.037; Adjusted R^2^ = 0.032; *F* = 6.953; *p* < 0.001					
Sleep/Rest scale score, model 2					
Ba, ppm	−0.001	0.001	−0.096	−2.244	0.025
Constant	81.016	0.705		114.982	<0.001
*N* = 546; R^2^ = 0.009; Adjusted R^2^ = 0.007; *F* = 5.035; *p* = 0.025					
Cognitive Fatigue score, model 1					
Health	3.590	0.531	0.278	6.755	<0.001
Number of after-school activities	1.262	0.418	0.124	3.017	0.003
Constant	66.107	1.855		35.635	<0.001
*N* = 540; R^2^ = 0.092; Adjusted R^2^ = 0.088; *F* = 27.158; *p* < 0.001					
Cognitive Fatigue score, model 2					
PM_2.5_ μg/m^3^, mean	−1.170	0.508	−0.098	−2.304	0.022
Constant	84.337	2.143		39.353	<0.001
*N* = 546; R^2^ = 0.010; Adjusted R^2^ = 0.008; *F* = 5.308; *p* = 0.022					

## 4 Discussion

Children spend significant part of their time in schools, mostly in their classrooms (10). Therefore, the quality of the air in the classrooms plays an important role in children’s cognitive performance, learning productivity and can be the reason for increased fatigue level (10, 11, 34). What is more, air pollution is a relevant risk factor for respiratory and other chronic conditions (8, 9, 35, 36). In our study we analyzed association of children self-reported fatigue and air pollutants in the classrooms. Fatigue is a complex phenomenon influenced by various factors (37), including indoor air quality (10, 11, 38). Our study confirms relationship between indoor air quality and fatigue in children. We revealed significant correlations between fatigue level and different air pollutants in school classrooms. Fatigue scores were lower which means higher lethargy in children who were exposed to higher concentrations of particulate matter, copper and vanadium. General Fatigue scale additionally correlated with tungsten and Cognitive Fatigue scale with lead. Sleep/Rest scale scores were lower, meaning higher fatigue in schools where arsenic, barium and strontium concentrations were higher. Although correlations were significant, but they were relatively weak which might be explained by presents of other confounding determinants of fatigue and small number of schools enrolled into the analysis.

Airborne particulate matter is one of the main air pollutants causing adverse health effects (10, 19, 39). It is well known that particulate matter causes oxidative stress and inflammation affecting key target—the mitochondria in lung tissue, brain and other organs (39, 40). This may result in different short and long-term outcomes: from reduced productivity and increased fatigue to mental and physical conditions such as depression, respiratory, cardiovascular and other chronic diseases (12–15, 38–40). It is also known, that the smaller the particles are, the more damaging effect they cause as they can penetrate more deeply and reach more mitochondria (39). This can also be seen in our study where higher fatigue level was independently associated with higher concentrations of PM_1_ and PM_2.5_, but not PM_10_. What is more, additional adverse mental health effects can be seen due to heavy metal deposition in prefrontal cortex (13, 41). In this study higher concentrations of vanadium and barium were independently related to higher fatigue level in children, which aligns with evidences that excessive intake of these elements can cause severe health issues, including cardiovascular, respiratory, digestive, and neurological dysfunctions, with barium poisoning causing hypokalemia and muscle weakness and vanadium exposure linked to kidney toxicity, neurotoxicity, and olfactory dysfunction (16, 42–44). In previous study we also found a linear relationship between respiratory morbidity in children and the concentration of vanadium in dust aggregates. This suggests that any concentration of vanadium inhaled by children can increase respiratory morbidity caused by viruses and bacteria (9). Based on this we might assume that higher levels of air pollutants can cause other health outcomes such as decrease in cognitive performance and increased level of fatigue.

The quality of the air in the classroom is determined by both, indoor and outdoor air pollution. The primary source of indoor air pollution in schools was identified as canteens (45). Outdoor air pollution is no less significant, especially in our study were schools enrolled represent urban living environment (46). Since Vilnius does not have significant industrial pollution sources, the primary contributors to air pollution are heating plants and traffic (27). Specific levels of outdoor air pollution near the studied schools may have been affected by the geographical characteristics of the area, as Vilnius is located in a highly hilly area near two rivers (47). Since the data for the study were collected during the winter, this may have resulted in higher levels of pollutant concentrations observed due to both increased outdoor emissions during heating season (27) and reduced ventilation caused by cold weather. The main sources of particulate matter in Europe are vehicle emissions, industrial and agricultural activities, residential heating and natural sources as crustal matter including dust from soils and wildfires (48, 49). Specifically, vanadium mostly comes from anthropogenic activities: the combustion of fossil fuels and emissions from vehicles and industrial production (9, 50). Barium comes from natural sources like soil erosion, which releases it into the air. It is also used in various industries, including oil and gas, medicine, and the production of paints, glass, rubber, ceramics, pesticides, and fuel additives (51).

An appropriate school ventilation system is essential for reducing both indoor and outdoor air pollution, as well as minimizing the risk of infection transmission (52, 53). Ventilation and strategies for reducing dust exposure as well as monitoring of indoor air quality would be key measures to improve quality of the air in the classroom, lessen its health impact and decrease fatigue in children accordingly (9, 13, 52). School administration, municipalities and public health specialist could be involved in these mitigation strategies. Parents or caregivers, as well as teachers, should be informed about possible air pollution at schools and its health effects on children (13).

Multivariate linear regression showed that only small proportion of variance in children fatigue can be explained by exposure to air pollutants at school. This can be explained by the fact that the study was conducted in regular schools where children spend their time and the quality of the environment is monitored and controlled. What is more, children fatigue is much more influenced by general health condition, social, environmental and lifestyle factors. Mean total fatigue score in this study exceeded 80 scores and was similar to scores of healthy populations in previous studies (54–56). Fatigue scores were higher compared to children with chronic conditions: neurofibromatosis (57), asthma (58), cancer, type 1 diabetes (56), and other diseases (22, 54, 55, 59). Less fatigued children showed better results at school and attended more after-school activities. Socio-economic conditions such as household income and deprivation, parental education level, housing quality, and access to healthcare can significantly affect a child’s health, stress and fatigue levels. In previous study we reported that lower socioeconomic status was related to higher fatigue among children with asthma (58). Similar results were found among children and adolescent’s females with physical disabilities (60). Despite this current understanding of how socio-demographic factors like socio-economic status and deprivation affect outcomes remains limited (57). In this study, we did not evaluate the respondents’ income, instead, we focused on housing type and whether the children had a separate room as indicators of housing and environmental quality. However, these factors were not found to be related to child fatigue. Family composition—another important determinant of fatigue in children. Children from single-parent families indicates higher levels of fatigue (58, 59). In this study we assessed only the number of siblings, but it was not related to fatigue scores. While we did not observe differences between boys and girls in our study, previous studies have reported gender differences, but the findings have been inconsistent (22, 59, 60). Although Smout with colleagues reported fatigue associations with BMI (61), in our study population this correlation was not found.

This study has both strengths and limitations. One of the advantages of our study is that micro-elemental analysis of air pollution in the classrooms was performed. Many studies focus on air pollution in terms of the concentration of particulate matter, O_3_, CO, CO_2_, NO_2_, VOCs as the main air pollutants (6, 10, 11, 19, 24, 62), but far fewer studies focus on the impact of individual elements on health and well-being. What is more, other studies analyzing dust composition (63, 64) collected dust from floors and windowsills and did not target to analyze the composition of the aerosol which is our study exclusivity. Another strength of our study is that validated methodology, the PedsQL Multidimensional Fatigue scale was used to assess fatigue in children. This instrument allows to compare results of our study with other studies, groups of population. Finally, there many studies analyzing fatigue in children with different chronic conditions (22, 54–61), but less studies focus on general population.

Our study has several limitations. First, a cross sectional type of the study allows identification of statistical associations, but it does not establish causality. The second limitation is that only subjective fatigue assessment was performed, and study would benefit from additional objective clinical and functional methods for fatigue measurement. The third limitation concerns the uncertainty regarding the duration of dust accumulation in each school. One of the weaknesses is that only 8 schools were enrolled in the study. Higher number of schools would be beneficial for statistical analysis and more associations could be found, especially in multivariate analysis. What is more, only self-reports were used for fatigue assessment and some studies indicate that inclusion of both, self- and proxy- reports could give additional insights (22, 58). Finally, only subjective health assessment was involved in the study. Inclusion of medical data with doctor-confirmed diagnosis would add value to the study. On the other hand, self-reported general health condition is a good indicator of mental health which is closely related to children’s fatigue level (65, 66). Therefore, further research analyzing the links between children’s fatigue and other factors including psychological well-being and lifestyle factors are being planned by our research group.

## 5 Conclusion

Fatigue in children is a complex phenomenon determined by many factors: health condition, socio-economic status, lifestyle factors, as well as environmental conditions, including air pollution. In this study we found that increased children’s fatigue was independently related to poorer general health conditions, lower results at school, and fewer after-class activities. Concentrations of particulate matter, barium and vanadium in the natural dust aggregates independently correlated with fatigue scores. These findings highlight the need for concrete public health actions, including regular and appropriate ventilation, standardized indoor air quality monitoring, and integration of air quality into school health policies. These measures, coordinated through collaboration among health authorities, educators, and caregivers, are essential to ensure a healthier, safer learning environment, reduce fatigue, and promote overall well-being in children.

## Data Availability

The raw data supporting the conclusions of this article will be made available by the authors, under reasonable request.
