# Supplementary material for: A cross-sectional analysis of air pollution in primary schools and children fatigue
Source: Front Public Health. 2025 Sep 1;13:1595089. doi: 10.3389/fpubh.2025.1595089 (PMC12434117; doi:10.3389/fpubh.2025.1595089)
Supplement: Supplementary file 1 [file Data_Sheet_1.docx]

**Annex 1.** General questionnaire used for a survey (English translation and original Lithuanian version)

**Dear Participant,**

Researchers at Vilnius University are conducting a study on fatigue among school-age children and aim to identify the main contributing factors.
We kindly ask you to complete this and the other provided questionnaires. Please answer every question in the questionnaire. Mark the appropriate answer with an X or circle it.

We sincerely thank you for your time.

**Vilnius University’s Researchers**

**1. Gender**

Boy

Girl

**2. Age_______ or birth date ____________________**

**3. Height _______cm**

**4. Weight _____kg**

**5. Do you have siblings?**

None

One

Two

Three and more

**6. Do you have your own room?**

Yes, I have a completely separate

Yes, with other siblings

I don‘t have

**7. How do you assess your health?**

Very good

Good

Average

Satisfactory

Poor

Very poor

8. **What after-school activities do you attend?**

Sports ...... (specify)

Swimming

Dance class

Singing

Art, ceramics

Other

I do not attend

**10. How do you assess your results at school?**

Good and very good

Average

Bad and very bad

**11. Where do you live?**

Own house

Terraced house

Apartment building (which floor……………)

**12.** Which part of Vilnius do you live in?

City center (Žvėrynas, Šnipiškės, Naujamiestis, Senamiestis)

Antakalnis

Žirmūnai

Šeškinė

Paneriai, Naujininkai, Vilkpėdė

Lazdynai, Karoliniškės

Justiniškės, Viršuliškės

Pilaitė

Pašilaišiai, Fabijoniškės

Verkiai

Rasos

Grigiškės

Other……………(specify)

Vilnius suburb (specify………………………….)

**13. How close to your home is the road where trolleybuses/buses run?**

very close

close

somewhat far

far away

**14. How close to your home is there a park or a forest?**

very close

close

somewhat far

far away

**15. How long do you usually stay outside per day during autumn?**

Up to 1 hour

1-2 hours

2-3 hours

3-4 hours

Other ….... (to fill in)

I don’t spend any time outdoors

**16. How long do you usually stay outside per day during winter?**

Up to 1 hour

1-2 hours

2-3 hours

3-4 hours

Other ….... (to fill in)

I don’t spend any time outdoors

**17. How long do you usually stay outside per day during spring?**

Up to 1 hour

1-2 hours

2-3 hours

3-4 hours

Other ….... (to fill in)

I don’t spend any time outdoors

**18. How long do you usually stay outside per day during summer?**

Up to 1 hour

1-2 hours

2-3 hours

3-4 hours

Other ….... (to fill in)

I don’t spend any time outdoors

**Thank you for completing the questionnaire!**

**Gerb. tyrimo dalyvi,**

**Vilniaus universiteto mokslininkai atlieka mokyklinio amžiaus vaikų nuovargio tyrimą ir siekia išsiaiškinti svarbiausius jį lemiančius veiksnius.**

**Labai prašome užpildyti šią bei kitas pateiktas anketas. Labai prašome atsakyti į kiekvieną anketos klausimą. Tinkamą atsakymą pažymėkite X arba apveskite.**

**Nuoširdžiai dėkojame už skirtą laiką.**

**Vilniaus universiteto mokslininkai**

**1. Lytis**

Berniukas

Mergaitė

**2. Amžius_______ arba vaiko gimimo data____________________**

**3. Ūgis_______cm**

**4. Svoris______kg**

**5. Ar turi brolių, sesių?**

Neturiu

Vieną

Du

Tris ir daugiau

**6. Ar turi savo atskirą kambarį?**

Taip, turiu visiškai atskirą

Taip, bet su kitais vaikais

Neturiu

**7. Kaip vertini savo sveikatą:**

Labai gera

Gera

Vidutinė

Patenkinama

Bloga

Labai bloga

8. **Kokius papildomus užsiėmimus lankai?**

Sportą ...... (nurodyti, kokį)

Plaukimą

Šokius

Dainavimą

Dailę, keramiką

Kitus būrelius

Nelankau

**10. Kaip vertini savo mokymosi rezultatus?**

Gerai ir labai gerai

Vidutiniškai

Blogai ir labai blogai

**11. Kur yra butas, kuriame Jūs gyvenate?**

Nuosavame name

Kotedže

Daugiabutyje .....(nurodykite kuriame aukšte)

**12. Kokioje Vilniaus vietoje yra butas, kuriame Jūs gyvenate:**

Centras (Žvėrynas, Šnipiškės, Naujamiestis, Senamiestis)

Antakalnis

Žirmūnai

Šeškinė

Paneriai, Naujininkai, Vilkpėdė

Lazdynai, Karoliniškės

Justiniškės, Viršuliškės

Pilaitė

Pašilaišiai, Fabijoniškės

Verkiai

Rasos

Grigiškės

Kita…….(nurodykite pavadinimą)

Vilniaus priemiestis…… (nurodykite pavadinimą)

**13. Kaip arti prie Jūsų namų yra kelias, kuriuo važinėja troleibusai arba autobusai?**

labai arti

arti

tolokai

toli

**14. Kaip arti prie Jūsų namų yra parkas arba miškas?**

labai arti

arti

tolokai

toli

**15. Kiek laiko vidutiniškai per dieną Jūs praleidžiate lauke rudenį?**

iki 1 val.

1-2 val.

2-3 val.

3-6 val.

kita .... (įrašyti)

nebūnu lauke visai

**16. Kiek laiko vidutiniškai per dieną Jūs praleidžiate lauke žiemą?**

iki 1 val.

1-2 val.

2-3 val.

3-6 val.

kita .... (įrašyti)

nebūnu lauke visai

**17. Kiek laiko vidutiniškai per dieną Jūs praleidžiate lauke pavasarį?**

iki 1 val.

1-2 val.

2-3 val.

3-4 val.

kita .... (įrašyti)

nebūnu lauke visai

**18. Kiek laiko vidutiniškai per dieną Jūs praleidžiate lauke vasarą?**

iki 1 val.

1-2 val.

2-3 val.

3-6 val.

kita .... (įrašyti)

nebūnu lauke visai

**Ačiū už atsakytus klausimus**
